# The effectiveness of psychological interventions alone, or in combination with phosphodiesterase-5 inhibitors, for the treatment of erectile dysfunction:A systematic review

**DOI:** 10.1080/2090598X.2021.1926763

**Published:** 2021-05-27

**Authors:** Sandrine Atallah, Asad Haydar, Teddy Jabbour, Peter Kfoury, Georgio Sader

**Affiliations:** a Department of Obstetrics and Gynecology, American University Beirut Medical Center (AUBMC), Beirut, Lebanon; b Faculty of Medicine, University of Balamand, Koura, Lebanon; c Faculty of Medicine, American University of Beirut, Beirut, Lebanon

**Keywords:** Erectile dysfunction, systematic review, psychological intervention, combined therapy, phosphodiesterase 5 inhibitors, cognitive behavioural therapy

## Abstract

**Objectives**: To highlight the efficacy of various psychological interventions (PI) when used in combination with, or in place of, phosphodiesterase-5 inhibitors (PDE-5i), as compared to the traditional treatment of men with erectile dysfunction (ED) with PDE-5i alone.

**Methods**: A comprehensive literature review for the years 2005–2020 via MEDLINE and PubMed. We included randomised controlled trials that compared the use of either PDE-5i alone, PI alone or a combination of PDE-5i and PI in the treatment of psychogenic ED. All studies included were performed in adults aged 19–55 years and were written in English.

**Results**: A total of 13 articles, with an overall sample of 597 men, were included in this systematic review. The results show that the combination of PI and PDE-5i was more effective than either PI or PDE-5i alone, on erectile function and long-term sexual satisfaction in men with psychogenic ED. Combined interventions were found to be significantly superior to medical treatment in seven studies and to PI alone in one study. In comparing PI to PDE-5i, two studies found PI to be significantly superior to PDE-5i use. In three other studies, PI was found significantly superior to no treatment at all, although some participants in the control group had taken PDE-5i.

**Conclusions**: The combination of PDE-5i with PI shows real promise for the treatment of psychogenic ED. However, no conclusions could be made about what PI is more promising than the other and larger studies are needed to confirm these initial findings.

**Abbreviations**: CBT: cognitive behavioural therapy; CBST: cognitive behavioural sex therapy; COVID-19: coronavirus disease 2019; ED: erectile dysfunction; EDITS: Erectile Dysfunction Inventory of Treatment Satisfaction; GPT, group psychotherapy: IIEF(-EF) (-OS) (-SD): International Index of Erectile Function (erectile functioning) (overall sexual satisfaction) (level of sexual desire); ITP: integrative treatment protocol; MHI: Mental Health Inventory; PDE-5i: phosphodiesterase-5 inhibitors; PI, psychological interventions; QoL: quality of life; RCT: randomised controlled trial; SHIM: Sexual Health Inventory for Men

## Introduction

Erectile dysfunction (ED) is a major health problem causing significant distress and negatively affecting the quality of life (QoL) of men and their partners [1]. According to the biopsychosocial model, ED aetiology is often multifactorial combining both psychogenic and organic components (mixed aetiologies) [2]. Psychological origins include performance anxiety, fear of failure, limiting sociocultural beliefs, inhibiting religious backgrounds, contextual and work-related stress, past sexual trauma, and relationship problems [3].

The prevalence of ED increases with age; however, its exact prevalence is difficult to estimate and varies from one study to another. Among young men (aged <40 years), reported ED prevalence varies from 1% to 10%. In slightly older men (aged 40–49 years), the estimates range between 2% and 15%. Among men aged 50–69 years, prevalence can reach 33% [4]. However, according to several studies, ED prevalence is increasing among younger men (aged <40 years) and is most probably being underestimated due to under-reporting [5]. Its actual prevalence among these men might be >20% [6]. It is also difficult to give precise estimates of the prevalence of psychogenic ED vs organic ED. For example, a Turkish study [7] found that 85.2% of young men in their sample (aged <40 years), presented a psychogenic ED, whereas older men had a 40.7% prevalence of psychogenic ED. While other studies had lower estimates, with an average prevalence of psychogenic ED of 40% among men aged <55 years [8,9]. Again, these differences can be explained by the multifactorial origins of ED on one hand, and by the heterogeneity of studies’ designs and methodologies on the other. Nevertheless, ED can lead to decreased self-esteem, emotional distress, higher risk of mental disorders (such as depression and anxiety), relationship problems, as well as low QoL. These consequences further impact erectile function, thus increasing fear of failure and trapping men in a vicious cycle of performance anxiety and sexual dysfunction [10].

The official launching and approval of sildenafil in 1998 drastically changed the management algorithm of ED. Psychological interventions (PI) were completely neglected as sildenafil was efficient among almost 70% of men with ED. However, one of the reasons why PI should be considered in the treatment of ED is the fact that despite the efficacy and safety profile of phosphodiesterase-5 inhibitors (PDE-5i), high rates (14–50%) of pharmacotherapy discontinuation are reported [11]. Incorrect use of PDE-5i (timing, dosage, lack of direct stimulation) or unrealistic expectations might be one of the explanations for treatment discontinuation [12,13]. The fact that medical treatments only manage the organic factors affecting erectile function and neglect the psychological factors in play, may also explain these rates [14].

The role of sociocultural factors in the management of psychogenic ED is also disregarded. Several studies reported a high prevalence of psychogenic ED among young Muslim men from the Middle East and the Indian subcontinent, due to misconceptions and sociocultural beliefs [15–18]. In these cultures, most men are reluctant to seek help, and when they do, they often await a medical diagnosis for their ED along with a quick treatment [19]. This is a matter that requires psychosexual education before prescribing any type of pharmacotherapy. Furthermore, sociocultural factors can lead to ED that is resistant to pharmacotherapy alone. For example, unconsummated marriage encountered mainly in conservative Middle Eastern societies and in some developing countries, is a frequent condition where newly married couples are unable to achieve penile-vaginal intercourse for variable periods despite desire and several attempts to do so [19,20]. The most common male reason for an unconsummated marriage is psychogenic ED also labelled as ‘honeymoon impotence’. In these scenarios, where addressing misconceptions and sociocultural beliefs is essential, combined therapy might achieve better results than PDE-5i alone, as the latter may aggravate the situation by putting more pressure on the spouses. Thus, implementing a combined approach integrating both medical and psychological interventions would increase ED treatment efficacy in the long term and decrease treatment discontinuation [21,22]*.

Finally, it is important to mention that the present review was conducted during a period of time where the coronavirus disease 2019 (COVID-19) pandemic has altered sexual medicine practice and sexuality in unprecedented ways. This unusual context might encourage for many reasons the use of PI in the management of psychogenic ED. Amongst those, the increased usage of telemedicine might increase the relevance, acceptance and need for internet-based interventions. Additionally, confinement and the disease in itself are both causes of psychological stress [21], which might precipitate previous subclinical psychogenic ED. Hence, personalised combined interventions may become even more essential in the near future to adequately address ED within a biopsychosocial frame [23].

## Aim of the review

The aim of the present review was to assess the comparative effectiveness of PI, PDE-5i and their combination in the management of ED.

## Methods

### Inclusion and exclusion criteria

A comprehensive literature search was performed to identify randomised controlled trials (RCTs) published from 2005 to and including July 2020. The selected RCTs compared the use of either PDE-5i alone, PI alone or a combination of PDE-5i, and PI in the treatment of psychogenic ED. PI comprised but were not limited to psychoeducation, cognitive behavioural therapy (CBT), mindfulness and internet-based therapy. Furthermore, all treatment modalities (individual, group or coupled) were included, as well as all types of PDE-5i.

The target population was focussed on young male adults (aged 19–55 years) who complained of psychogenic or mixed (multiple factors) ED. Thus, studies that involved only men aged >55 years or men complaining solely of organic ED were excluded.

The main outcome measures chosen were the change in the ED status, as measured by validated questionnaires (e.g. International Index of Erectile Function [IIEF]), and patients’ treatment and sexual satisfaction. Long-term outcome measures were also based on ED status and patients’ satisfaction on follow-up assessment 6–18 months later depending on the study.

### Search strategy

Studies were identified by searching through the electronic databases PUBMED and MEDLINE (Ovid) by following through the following algorithm:

1. (erectile dysfunction*) OR (impoten*) OR (erection failure) OR (male sexual dysfunction)
2. (psychotherap*) OR (psychosocial intervention) OR (psychoeducation) OR (coping skills) OR (brief motivational counseling) OR (sexual therapy) OR (anxiety management training) OR (marital therapy) OR (group therapy) OR (cognitive therapy) OR (behavio* therapy) OR (general counseling) OR (psychodynamic therapy) OR (supportive therapy) OR (mindfulness) OR (interpersonal therapy) OR (cognitive Behavioral therapy) OR (individual therapy) OR (couples therapy) OR (internet based therapy) OR (combined therapy) OR (Acceptance and Commitment Therapy)
3. Sildenafil OR Tadalafil OR Vardenafil OR Avanafil OR (PDE5 inhibit*) OR (phosphodiesterase 5 inhibit*)
4. *2 AND 3*
5. *1 AND 4*

*Filters: Randomized Controlled Trials, Humans, Adult: 19–55 years, from 2005–2020*

### Selection of studies

After searching the databases, 264 articles that matched this review’s search strategy were identified. After eliminating duplicates and adding articles from other sources, 262 articles were screened based on title and abstract. The articles that had the potential of being included in this study were obtained in full-text in order to further review them for eligibility based on the inclusion and exclusion criteria. The references of the selected articles were then crosschecked. Finally, 33 articles were selected to be assessed as full-text articles. After that, 20 studies were excluded: six studies were not RCTs, 12 did not evaluate a psychological intervention, one evaluated organic ED, and one did not study our targeted population. This systematic review was conducted according to the Preferred Reporting Items for Systematic Reviews and Meta-Analyses (PRISMA) guidelines.

**Figure 1.**
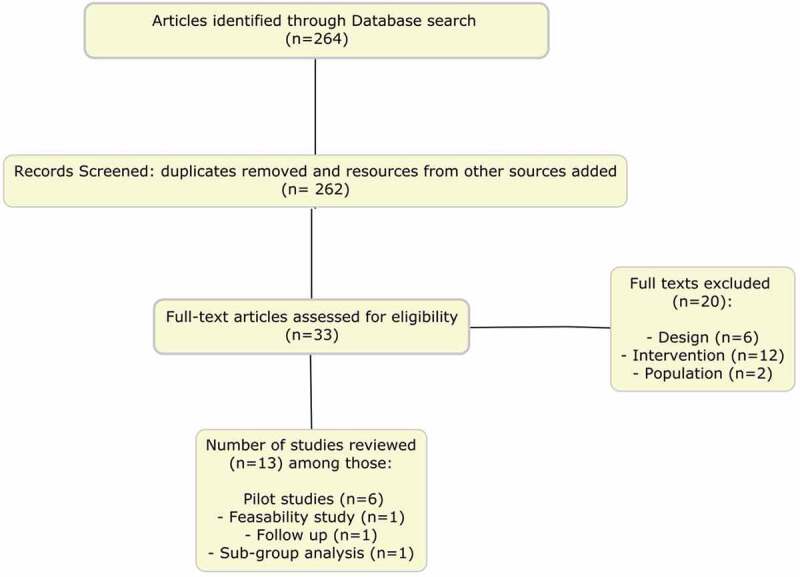
Flowchart of article selection

A flowchart describing the results of the literature search and the number of included studies is shown in Figure 1.

### Studies’ description

This review assembled 13 articles among those six pilot studies, one feasibility study, one follow-up study and one sub-group analysis. The most common types of PI were reviewed and these included: individual CBT/cognitive behavioural sex therapy (CBST), group therapy and counselling, couple CBST, and internet-based interventions. Mindfulness and psychoeducation could not be included due to lack of studies meeting the selection criteria

## Results

A total of 13 studies published between 2005 and 2020 were included in the review (Table 1) [24–36]. The global sample included 597 men (ranging from 12 to 117 men/study), and included men from different cultures. Three studies were run in Brazil, three in Pakistan, two in the USA, two in Australia and one in each of Italy, Sweden and the Netherlands. All studies were published in English.

**Table 1.** Studies’ characteristics

Reference	Methods	Participants	Interventions	Outcomes	Limitations	Clinical implications	
Individual CBT							
Khan *et al*. 2017 [24]	Allocation: quasi systematised 2 groups: - CBT group (CBT + PDE-5i) - Control group (PDE-5i only)	60 men Mean age 26.8 years Mean age Group I, 28 years; and Group II, 25.6 years Diagnosis: Psychogenic ED	-CBT + PDE-5i (*n* = 30) -Control: PDE-5i alone (*n* = 30) Duration: 10 weeks	-IIEF -MHI (anxiety and depression scores)	-Sample size -short duration -PDE-5i use not monitored -self-selected sample -Sample not fully systematised -No CBT-alone group -Absence of a placebo + CBT group -No evaluation or inclusion of the female partner		-Superiority of combined interventions -Improvement of anxiety scores -Cultural added value
Khan *et al*. 2019 [25]	Follow-up after 18 months - CBT group - Control group	20 men Age 27.2 years Mean age Group I, 26.2 years; and Group II, 28.2 years Diagnosis: Psychogenic ED	Follow-up after 18 months of - CBT + PDE-5i (*n* = 10) - Control: PDE-5i alone (*n* = 10)	-IIEF -MHI (anxiety and depression scores)	-Sample size -No evaluation or inclusion of the female partner		-Superiority of combined interventions on long term improvement -Cultural added value
Bilal 2020 [26]	Feasibility study Allocation: randomised, sequential 2 groups - CBST group (CBST alone) - Sildenafil 50 mg only	28 men Mean age 31 years Diagnosis: psychogenic ED (non-organic ED)	-CBST twice weekly (*n* = 13) 4 weeks if mild 6 weeks if mild to moderate 8 weeks if moderate 12 weeks if severe -Sildenafil 50 mg on-demand Duration: 12 weeks	-IIEF-5 -DASS-21	-Sample size -Short duration -No investigative techniques to rule out the organic aetiology -Problematic study design: duration of CBST different according to ED severity -Absence of control group (no treatment or placebo) -Absence of a placebo + CBST group -IIEF-5 used and not IIEF -Frequency of sexual intercourse not assessed -No assessment of sexual enjoyment and pleasure as outcome -No evaluation or inclusion of the female partner		-Same efficacy of CBST compared to sildenafil -Improvement of anxiety and depression scores with CBST -Cultural added value
Group interventions							
Melnik and Abdo 2005 [27]	Allocation: randomised 3 groups: -Group I: group therapy + 50 mg sildenafil citrate - Group II: 50 mg sildenafil citrate alone -Group III: group therapy alone	22 men Mean age 39.74 years Group I, 40.1 years; Group II, 36.2 years; and Group III, 42.75 years Diagnosis: psychogenic ED	-Group I: group therapy (weekly sessions for 6 months) + 50 mg sildenafil citrate orally on demand (*n* = 8) -Group II: 50 mg sildenafil citrate orally on demand (*n* = 6) -Group III: group therapy (weekly sessions) (*n* = 8) Duration: 6 months FU at 3 months	-IIEF -Presence of ED	-Small sample size -Strict inclusion criteria (only 10% of screened patients were included) hence questioning sample representativeness -Absence of a placebo + GPT group -No data collected from female partners	-Superiority of psychotherapy and combined interventions -Superiority of psychotherapy and combined interventions on long-term improvement	
Abdo *et al*. 2008 [28]	Allocation: randomised 3 treatments groups: -Group I: counselling - Group II: sildenafil alone -Group III: sildenafil + counselling	110 men Mean age 47.6 years Mean age Group I, 46.7 years; Group II, 48.9 years; and Group III, 47.2 years Diagnosis: psychogenic and mixed ED	-Group I: group counselling (once a week) (*n* = 37) -Group II: sildenafil 50 mg up to 100 mg and up to 2 tablets/week (*n* = 40) -Group III: weekly group counselling + sildenafil (50 mg up to 100 mg, up to 2 tablets/week) (*n* = 40) Duration: 3 months	-MSQ -SHIM	-ED of mixed aetiologies -Absence of a placebo + counselling group -No data collected from female partners -Self-selected men highly motivated to address their longstanding ED problem -Short treatment outcome assessment period	Superiority of combined intervention and PDE-5i	
Melnik *et al*. 2012 [29]	Allocation: randomised 3 groups: -Group 1: group therapy alone - Group 2: 50 mg sildenafil citrate alone -Group 3: group therapy alone + 50 mg sildenafil citrate	22 men Mean age 40 years Mean age Group 1, 42.7 years; Group 2, 36.8 years; and Group 3, 40.1 years Diagnosis: psychogenic ED	-Group 1: group therapy alone (weekly sessions) -Group 2: 50 mg sildenafil citrate orally on demand (*n* = 6) -Group 3: GPT + 50 mg sildenafil citrate orally on demand (*n* = 8) Duration: 6 months FU at 3 months	-EDITS	-Small sample size -Only EDITS was used without IIEF -Strict inclusion criteria (only 10% of screened patients were included) hence questioning sample representativeness -Absence of a placebo plus counselling group	-Superiority of psychotherapy and combined interventions -Superiority of psychotherapy and combined interventions on long-term improvement	
Couple interventions							
Banner and Rodney 2007 [30]	Pilot Study Allocation: randomised 2 groups: -A: sildenafil alone -B: ITP: sildenafil + CBST	53 couples Mean age 56.8 years Mean age of men in Group A, 56.3 years; and Group B: 57.3 years Diagnosis: psychogenic ED	-Group A: sildenafil alone. If ‘no success’, additional CBST (*n* = 24) -Group B: ITP: sildenafil + CBST (*n* = 29) Duration: 8 weeks	-IIEF (M + W) -RDAS (M + W) -BDI (M) -BAI (M)	-Pilot study -Small sample size -Mean age >55 years -Problematic study design: Group A received the same treatment as Group B in the case of failure after the first 4 weeks -No psychological intervention alone group -Short treatment outcome assessment period -Absence of a placebo plus ITP group -No assessment of female sexual function	Higher rates of clinical success of combined interventions	
Aubin *et al*. 2009 [31]	Pilot study Allocation: Randomised 2 groups: -VO: sildenafil alone -VST: sex therapy + sildenafil	44 couples Mean age of men 52.71 years Mean age of men in VO group: 50.7 years and women 49.0 years Mean age of men in VST group: 54.4 years and women 50.9 years Diagnosis: ED of mixed aetiologies	-VO: Viagra only (*n* = 20) -VST: Viagra + sex therapy (*n* = 24) Duration: 12 weeks of treatment FU: 2 months	-IIEF -FSFI -PAIR (two emotional and sexual intimacy scales) -NTDSQ (four sexual cognition scales) -DAS -EDITS -SRQ	-Pilot study -Small sample size -Age group -Inclusion of participants with organic risk -Absence of a placebo plus ST group -No ST-only group -Sample not representative of the general population: 1-Subjects self-selection: self-selected couples highly motivated to address their longstanding ED problem 2-High level of education with a difference between the two groups: higher levels of education of VST group 3-Couples in stable, long-term relationship presenting with higher than average couple satisfaction, intimacy, and adaptation. factors for ED -Short treatment outcome assessment period	-Combined intervention associated with the maintenance of gains in most domains of sexual function, cognition, and intimacy -Superiority of the combined intervention in the maintenance of the positive erectile results compared to the results obtained with the pharmacotherapy alone. -Superior male and female treatment satisfaction in the combined intervention group	
Boddi *et al*. 2015 [32]	Pilot Study Allocation: randomised 2 groups: -A: VARD alone -B: VARD + CBST	30 couples Mean age of men 45.8 years Mean age in Group A: men 46.5 years, women 42.7 years; and in Group B: men 44.6 years, women 39.3 years Diagnosis: ED without a significant organic component	-Group A: VARD alone (*n* = 19) -Group B: VARD + CBST (*n* = 11) Duration: 10 weeks	-IIEF -FSFI -ISS	-Pilot study -Small sample size -No CBST alone group -Absence of a placebo plus counselling group -Short treatment outcome assessment period	-Superiority of combined interventions in the maintenance of the positive erectile results initially obtained with the pharmacotherapy alone. -Only combined interventions improved couple sexual satisfaction and female sexual function	
Internet based interventions							
McCabe *et al*. 2008 [33]	Pilot study Allocation: Non-randomised 2 groups: -Treatment group -Control	31 couples Age ? Diagnosis: ED	-Treatment group: internet-based therapy (rekindle) cognitive-behavioural and sexual therapeutic approach (sensate focus, communication exercises, and e-mail contact with therapist) (*n* = 12) -Control group (*n* = 19) Duration: 10 weeks FU: 3 months	-IIEF -KMSS -ISS -SEAR	-Pilot study -Small sample size -No data on type of pharmacological treatment, frequency, or dosage -High drop-out rate in the treatment group -Questionable reliability of diagnoses (partly medical diagnosis and partly self-reported) -No data collected from female partners -Short treatment outcome assessment period	-Improvement of EF after internet-based sex therapy compared to control group	
McCabe and Price 2008 [34]	Pilot study Allocation: Sub-group analysis 2 Groups: -Rekindle alone -Rekindle +PDE-5i	*N* = 12 couples Age = mean 54.5 years Age = mean Group 1: 56.6 years Group 2: 53.0 years Diagnosis: ED	-Internet-based therapy Rekindle (*n* = 5) -Internet-based therapy rekindle + PDE-5i (*n* = 7) Duration: 10 weeks FU: 3 months	-IIEF -KMSS -ISS -SEAR -WHOQOL-BREF	-Pilot study -Very small sample size -Age group -Difference in severity of ED: longer duration and higher frequency of ED before treatment in men taking PDE-5i -No PDE-5i alone group -Absence of a placebo plus Rekindle group -No control for factors such as type of pharmacological treatment, frequency, or dosage -Inclusion of participants with organic risk factors for ED -short treatment outcome assessment period	-Similar impact of internet based sex therapy regardless of whether or not oral treatment for ED had been used.	
van Lankveld *et al*. 2009 [35]	Pilot study Allocation: Randomized 2 Groups: -Direct treatment -Waiting list	*N* = 98 58 men with ED (40 men with PE) Age = mean 43.3 years Diagnosis: Self-reported ED (and self-reported PE)	-Direct treatment group: Internet-based sex therapy (Masters and Johnson approach, sensate focus, cognitive interventions, and psychoeducation) (3 months, FU at 3 and 6 months after treatment) (*n* = 30) -Waiting list control group (waiting 3 months, treatment for 3 months, FU at 3 months) (*n* = 28) Duration: 9 months	-IIEF-EF, IIEF-SD -IIEF-OS -SEAR-CONF	-Pilot study -Sample not representative of the general male population (75% highly educated) -Elevated frequency of emails exchange between therapist and participants -Pharmacological treatment recommended to 25% of participants (unclear if efficacy rates due to sex therapy or pharmacotherapy) -Study funded by pharmaceutical industry	-Improvement of EF after internet-based sex therapy -Internet-based therapy for ED superior to waiting list	
Andersson *et al*. 2011 [36]	Allocation: Randomised 2 groups: -ICBT -Control: online discussion group	*N* = 76 men Age = mean 56.51 years Age = mean ICBT: 57.62 years Control: 55.50 years Diagnosis: ED of mixed o rigins	-Treatment group: ICBT (N = 37) -Control group: online discussion group (*n* = 39) After 7 weeks, the control group received ICBT Duration: 7 weeks of treatment FU at 6 months	-IIEF-5 -RAS -BDI -BAI -WHOQOL-BREF	-Sample size -Mean age of participants>55 years -Medical conditions self-reported -ED of mixed origins: Psychogenic ED not used as an inclusion criterion -The choice of IIEF-5 as primary outcome measure does not provide a full clinical picture of the ED diagnosis -No control for factors such as type of pharmacological treatment, frequency, or dosage -High dropout rate: The number of completed modules was low maybe because of the short therapy duration -Lack of experimental control regarding the 6-month follow-up data, which in this study were compared with the control group post-treatment data	-Improvement in erectile performance that increase at follow up	

BAI: Beck Anxiety Inventory; BDI: Beck Depression Inventory; CBT: cognitive behavioural therapy; CBST: cognitive behavioural sex therapy; DAS: Dyadic Adjustment Scale; DASS: Depression Anxiety Stress Scale; ED: erectile dysfunction; EDITS: Erectile Dysfunction Inventory of Treatment Satisfaction; EF: erectile functioning; FSFI: Female Sexual Function Index; FU: follow-up;; GPT: Group Psychotherapy; ICBT: Internet-delivered cognitive behavioural therapy; IIEF: International Index of Erectile Function; ISS: Index of Sexual Satisfaction; ITP: integrative treatment protocol; KMSS: Kansas Marital Satisfaction Scale; MHI: Mental Health Inventory; MSQ: Male Sexual Quotient; NTDSQ: Negative Automatic Thoughts During Sex Questionnaire; OS: Overall Sexual Satisfaction; SEAR: Self-esteem and Relationship Questionnaire; SHIM: Sexual Health Inventory for Men; PAIR: Personal Assessment of Intimacy in Relationships; PDE-5i: phosphodiesterase type 5 inhibitors; PE: premature ejaculation; RDAS: Revised Dyadic Adjustment Scale; RAS: Relationship Assessment Scale; SD: sexual desire; SEAR-CONF: Confidence subscale (four items) of the Self-Esteem and Relationship Questionnaire; SRQ: Self-Report Evaluation Questionnaire of Treatment Outcome and Satisfaction; VARD: vardenafil orodispersible tablet; VO: Viagra Only; VST: Viagra + Sex Therapy; WHOQOL-BREF: World Health Organization Quality of Life-BREF.

Concerning PI, three studies used individual CBT/CBST [24–26], three studies employed group therapy-based interventions [27–29], three studies used couple-CBST [30–32], and four studies employed internet-based CBT [33–36]. PI duration ranged from an average of 4–12 weeks.

Regarding PDE-5i, six studies used sildenafil [25,26,28–31], one used vardenafil [32], and six did not specify the drug used by the patients [24,25,33–36].

A total of 12 studies reported change of ED status as the main outcome, while one study only used the IIEF to select the sample and then used the Erectile Dysfunction Inventory of Treatment Satisfaction (EDITS) as the outcome measure after treatment [29]. Among the 12 studies, several IIEF versions were used (either full, or IIEF-5/Sexual Health Inventory for Men [SHIM], IIEF/erectile functioning [IIEF-EF]/level of sexual desire [IIEF-SD]/overall sexual satisfaction [IIEF-OS]). One study also asked the female partner to complete the IIEF [30]. Furthermore, two studies assessed female sexual function [31,32] and two evaluated treatment satisfaction [29,31]

Results of the studies included in this review are organised according to the PI used.

### Combined PDE-5i with individual CBT

A study conducted by Khan *et al*. [24] consisted of 60 participants (mean age 26.8 years), recruited through several hospitals in Pakistan, who were assigned to either combined intervention (PDE-5i and CBT; *n* = 30) or monotherapy (PDE-5i alone; *n* = 30). At the end of the intervention period (10 weeks), there was a statistically significant difference in the IIEF scores between the two groups, with patients assigned to the combined therapy scoring higher in every category of the IIEF, except in orgasmic function. Furthermore, the Mental Health Inventory (MHI) anxiety scores showed a decrease in both groups, the difference being greater in the combined therapy group.

Khan *et al*. [25] conducted a follow-up study where they re-assessed the participants of the previous study on their erectile functioning 18 months later. A total of 20 men agreed to participate in this follow-up, 10 from the combined therapy group and 10 from the monotherapy group. The results showed that participants from the combined group still scored significantly higher on the IIEF than the monotherapy group. The MHI anxiety scores remained the same in both groups in the post-assessment period.

These two studies demonstrate the benefit of a combined approach to ED as compared to pharmacological intervention alone both in the short and long terms. Limitations included a small sample size, no monitoring of PDE-5i use, self-selecting and not fully systematised sample. In the follow-up study, an additional limitation is the large number of participants from the original study who dropped out, which compromises the validity of the study.

The most recent study comparing CBST to PDE-5i is that of Bilal [26]. A total of 32 men (mean age 31 years) referred from sexual health clinics in Pakistan, participated in the study for a period of 12 weeks. Half of the patients were allocated to the CBST arm (*n* = 16) and the other half to the sildenafil citrate 50 mg arm. There was no significant difference in the IIEF-5 scores for the CBST and sildenafil citrate groups. However, participants in the CBST intervention showed a significant reduction in anxiety scores, whereas anxiety scores were unchanged for the participants given sildenafil citrate. Limitations of the study included the small sample size and the short duration of the intervention. Furthermore, an organic aetiology was not properly ruled out. The participants in the CBST group were given different durations of treatment, which is a protocol that lacks a scientific basis.

### Group therapy and combined PDE-5i with group therapy

Melnik and Abdo [27] tested the difference in treatment efficacy between pharmacotherapy alone, psychotherapy (theme-based group psychotherapy) alone, and combined pharmaco-psychotherapy. A total of 22 men (mean age 39.74 years) with psychogenic ED were divided in three groups and administered the IIEF before treatment, immediately after treatment (6-month duration), and at a 3-month follow up. Even though all three treatment approaches led to an improvement in erectile function, the improvement was significantly higher in the PI alone and combined-treatment groups than in the PDE-5i alone group. There was no significant difference in improvement between PI alone and combined treatment. The study also found continued improvement of erectile function after discontinuation of group therapy, which indicates better long-term results. Limitations of the above study include its small sample size, very strict inclusion criteria, a high dropout rate, and the lack of a control group and placebo + PI group.

The same Brazilian team [28] conducted a study on 110 men (mean age 47.6 years) with psychogenic and mixed ED that were randomised into three different groups: counselling alone, sildenafil alone, and sildenafil with counselling. Significant improvement in erectile function and sexual satisfaction was found in all three groups after 3 months. Furthermore, combined treatment and sildenafil alone were significantly more effective than psychotherapy alone. Despite its large sample size, this study poses several limitations. These include the inclusion of ED of various aetiologies, which could explain the limited efficacy of psychotherapy alone. Plus, PDE-5i intake was not monitored.

A third study by Melnik *et al*. [29] compared the efficacy of three treatments modalities for psychogenic ED by measuring ED treatment satisfaction. This randomised clinical single-blind trial included 22 men (mean age 40 years) with psychogenic ED that were randomised to receive for 6 months: group psychotherapy (GPT) and 50 mg of sildenafil on-demand (Group I); or 50 mg of sildenafil on-demand exclusively (Group II), or GPT exclusively (Group III). Changes in score from baseline for three questions of the EDITS were evaluated at the endpoint and at the 3-month follow-up. Treatment satisfaction was significantly higher in the psychotherapy and combined-treatment groups than in the PDE-5i group. The limitations of this study include a very small sample size, strict inclusion criteria questioning the sample’s representativeness, and the absence of a control group and a placebo plus counselling group. Here again, the precise intake of sildenafil was not monitored. It is also worth noting that EDITS was used without the IIEF as an objective measure of ED.

### Combined PDE-5i with couple therapy

In a pilot study by Banner and Anderson [30], 53 couples with ED were studied (mean age of the men: 56.8 years), with the objective of evaluating an integrative treatment protocol (ITP) combining CBST with sildenafil. Patients were divided into two arms: Group A (*n* = 24) received sildenafil only, while Group B (*n* = 29) received ITP. At the end of the 8-week period, both groups showed a significant increase in IIEF-EF scores, which almost doubled from baseline. The study found that combined therapy had higher rates of clinical success, and was efficient when ED was refractory to pharmacotherapy alone. Limitations include a small-scale pilot study and a problematic study design, as Group A received the same treatment as Group B in case of failure after the first 4 weeks. The mean age of the male participants was ~56 years, which is older than the target age for this review. Furthermore, the ITP group was not compared to a CBST-alone group, or a placebo. Also, while female partners were assessed for IIEF, their own sexual function and satisfaction were not measured

In their study, Aubin *et al*. [31], sought to examine the effectiveness of a drug only vs a combined treatment approach on ED. The population included 44 couples between the ages of 20 and 80 years (mean age of the men: 52.71 years) in a stable relationship. A total of 20 couples were assigned to the Viagra only group and 24 couples to the Viagra plus sex therapy group. Assessment of response was performed: at 4 weeks after first treatment, last week of treatment (12 weeks) and 2-months after treatment. The combined intervention was significantly superior in the maintenance of the positive erectile results compared to the results obtained with the pharmacotherapy alone. Both partners had a higher treatment satisfaction in the combined therapy group. Limitations of the study include a small sample size, lack of control group and short follow-up period. This study had multiple confounding factors by including patients with organic risk factors for ED and selection bias of predefined couple dynamics not representative of the general population.

A more recent pilot study by Boddi *et al*. [32] recruited 30 couples (mean age of the men: 45.8 years) complaining of non-organic ED. A total of 19 couples were assigned to the vardenafil group and 11 to the vardenafil + 10 weeks of CBST group. This study showed that even though pharmacological therapy initially improved ED independently of any adjunct therapy, the addition of CBST increased the likelihood that this improvement would be maintained in the long term. Only combined interventions improved couple sexual satisfaction and female sexual function. Limitations include a small sample size pilot study and a short treatment-outcome assessment period. Additionally, the combined therapy group was not compared to a CBST-alone group.

### Internet-based intervention and combined PDE-5i with an internet-based intervention

McCabe *et al*. [33] enrolled 31 men with ED and their partners; of which 12 participated in a 10-week internet-based programme and a control group of 19 male patients that received no treatment. All men completed questionnaires at pre-test, post-test, and 3 months after completion of the 10-week programme. The internet-based programme resulted in improvement in erectile function, but no significant change in the remaining studied areas. Gains during the therapy period were shown to be largely maintained at follow-up. It is important to mention that in this study, men who were already on oral ED medications were not asked to discontinue their prior treatments, but incorporating new medications during the study time-period was prohibited.

In a second paper, McCabe and Price [34] ran a sub-group analysis of their first study]33[. The authors aimed to compare the efficacy of internet-based sex therapy alone with the efficacy of combined PDE-5i and internet-based sex therapy among the treatment group of their RCT (*n* = 12). The 12 couples (mean age 54.5 years) were distributed into one of the two treatment approaches: seven men had had the combined treatment and five had undergone psychological treatment alone. Participants who received internet therapy combined with medication reported similar results as participants who received internet therapy alone. The initial study was a non-randomised pilot trial that had a small number of participants and a high dropout rate. In addition, the follow-up period was relatively short, thus limiting adequate analysis of the results in the long term. It is also important to note that there were no data on the type, dosage and usage of ED medication. Although the treatment targeted couples, the female partners’ sexual function and satisfaction were not evaluated. Finally, among the men included, many were older than the review target population (i.e. aged <55 years) and had organic risk factors for their ED.

Van Lankveld *et al*. [35] studied internet-based therapy for both ED and premature ejaculation (PE) in a RCT pilot trial. Their study enrolled 98 heterosexual men, of which 58 had ED (mean age: 43.3 years). The ED group was further divided into a direct treatment (*n* = 30) and a ‘waiting-list’ control group (*n* = 28). The total duration of participation in the study was 9 months and included 3 months of treatment for both groups (the control group receiving treatment after 3 months of waiting), a first assessment at 3 months, two follow-up assessments at 3 and 6 months for the first group, and one follow-up assessment at 3 months for the second group. The study found improvements in erectile functioning and overall sexual satisfaction in both control and treatment arms. However, more notably, *post hoc* univariate tests showed greater improvement in the treatment group when the measured outcomes were sexual desire and sexual self-confidence in comparison to the waiting-list control group. As for the maintenance of the outcomes, within the follow-up phase, individuals improved significantly from pre- to post-treatment. This study had several limitations. It was a pilot trial with a sample that was not representative of the general male population, as a high percentage of educated males were included. The efficacy of the PI might be overestimated due to the intake of pharmacotherapy that was advised to 25% of the participants without monitoring. Lastly, the study was funded by the pharmaceutical industry, which could be considered a conflict of interest.

Andersson *et al*. [36] also aimed to study the effects of internet-based CBT (ICBT) on individuals with ED. A total of 76 men (mean age: 56.51 years) with mixed aetiologies of ED were allocated randomly into two groups: one receiving ICBT and a control group that had access to online discussions. The treatment was administered through a 7-week internet-based programme with e-mail therapist support. After 7 weeks from initiation of the trial, the control group received ICBT. Both groups were re-assessed at a 6-month follow-up. The study showed a significant improvement in the erectile function of the ICBT group in comparison to the control group. The results analysis also showed an improvement up to the 6-month follow-up. However, this study had several limitations. The mean age of the participants was slightly higher than the target population of this review. Plus, men with ED of mixed origins (organic and psychological) were included. Participants were also allowed to take pharmacotherapy and this intake was not monitored. It is difficult to interpret the results at follow-up because of the lack of experimental control.

## Discussion

The present review indicates that the combination of PI and PDE-5i has more effect when compared with either PI or PDE-5i alone on erectile function and long-term sexual satisfaction in men with ED. Combined interventions were found to be significantly superior to medical treatment in seven studies and to PI alone in one study. One study reported no significant differences between combined treatment and PI.

In comparing PI to PDE-5i, two studies found PI to be significantly superior to PDE-5i use. In three other studies, PI was found to be significantly superior to no treatment at all, although some participants in the control group had taken PDE-5i.

Overall, the studies included in the present review had some common limitations. All except five studies had a sample size of <50 participants, which does not yield enough statistical power. Furthermore, in most studies, organic ED was not properly ruled out. Therefore, because of the inclusion of participants from an older age group, it is possible that some of the participants in these studies had organic or mixed aetiology ED and were not the best candidates to receive PI. Also, despite the fact that several studies included the female partner in the PIs, most studies did not evaluate female sexual function and satisfaction.

Despite these limitations, the present review confirms the conclusions of previous reviews [37,38] with a superior effectiveness of combined interventions compared with PDE-5i alone. This poses several opportunities and challenges when tackling combined therapy:

1. In cases of psychogenic ED, combined approaches allow the management of psychosocial factors that cannot be tackled by PDE-5i alone. Despite their limitations, three studies in the present review [24–26] found that combining individual CBST with PDE-5i was not only superior to PDE-5i when measuring ED improvement, but also in reducing anxiety levels.
2. Combined therapy can also increase treatment satisfaction and results in better long-term improvement. Three trials [27–29] reported the superiority of group interventions in combination with pharmacotherapy when compared with PDE-5i alone. Among those, two trials [27,29] even demonstrated that group therapy alone had better outcomes on ED and treatment satisfaction than PDE-5i alone. Three trials [30–32] found that combined interventions using couple sex therapy had higher rates of clinical success. One of these studies [31] reported better long-term effects, along with a higher treatment satisfaction. Two of these three trials [31,32] also found better female sexual and treatment satisfaction. These results suggest that a combined approach that includes the female partner within the management plan is more effective than pharmacotherapy alone. Unfortunately, no trial compared different combined approaches (individual CBST vs couple sex therapy).
3. Three studies and one sub-group analysis suggested that internet-based PIs could be beneficial in the management of mixed ED. It is true that these studies present numerous limitations. However, in the context of the COVID-19 pandemic, recent papers are encouraging the promotion of internet interventions in the field of sexual medicine [39,40]. Internet-based sex therapy not only respects social distancing, but also can easily and cost-effectively reach isolated populations with scarce sexual health resources across borders. Plus, internet-based sex therapy can provide psychological counselling to men who would like treatment for ED, but are too afraid or shy to solicit support in person [33], especially in a cultural context where both sexual health and mental health are considered as taboo topics [33].
4. As mentioned in the introduction, one of the challenges when it comes to PI and combined interventions is their implementation in non-Western cultures with a culture-sensitive approach [19]. Despite their distrust in PDE-5i, a lot of non-Western men prefer pharmacotherapy to psychotherapy [41]. Moreover, because these patients fail to understand the rationale behind PI, many do not adhere to the treatment [42,43]. However, the present review included two studies and one follow-up study from non-Western cultures [24–26]. These studies had promising results suggesting that PI and combined interventions could be successfully used in the management of psychogenic ED in a non-Western cultural context. It is true that these studies had several limitations and that due to cultural factors, female partners were not included. Thus, developing culture sensitive PI that would offer culturally ‘acceptable’ care can optimise combined interventions and increase their effectiveness.

### Limitations and recommendations for future studies

Challenges of the present review were the lack of studies found on various topics most notably mindfulness and psychoeducation, such that no studies were found about these two interventions that met the selection criteria. Due to the limited number of studies (13), the inclusion criteria were not strictly adhered to. Therefore, within the present review, some studies included participants of mixed aetiology ED, and with an age range that was wider than originally intended. For the same reasons, the present review included six pilot studies, one feasibility study, and one sub-group analysis. As for the outcomes, as no meta-analysis was conducted, there was no conclusion reached regarding the best treatment method. This issue was further highlighted due to the lacking and contradictions in some of the study designs. Another important limitation was the rather low methodological quality of most studies (sample size, sample selection, assessment tools, monitoring of PDE-5i intake), which makes definite conclusions difficult to draw.

Therefore, it is recommended for future studies to evaluate each PI with proper blinding (control group or placebo group) and aim to compare the different PI with each other using sound research designs (RCT, larger samples, longer follow-up). With the lack of current studies regarding psychoeducation and mindfulness, it is recommended to develop studies assessing these treatment modalities. It is also recommended to include, in future RCTs, assessment of sexual and treatment satisfaction of partners. Furthermore, as combined interventions seem a more efficient treatment approach than single treatment, such trials should be prioritised in order to come to stronger conclusions about their effectiveness

Finally, based on the findings of the present review, it is recommended that clinicians use any form of CBT/CBST (group or couple therapy over individual therapy) for men with ED. As most studies showed that combined PI and medical treatment was more effective than psychological or medical treatment alone, it is also recommended that clinicians use PI in addition to pharmacotherapy

## Conclusion

Combined interventions address the relevant medical and psychosocial origins of ED. Most studies in the present review demonstrated superiority of combined treatment when compared to single approach interventions (PI alone, PDE-5i alone). The promising findings and the limitations of these studies warrant the need for further RCTs on PI and combined interventions in the treatment of ED. In view of the positive results, it is recommended that healthcare providers include in their management of ED combination or integrated treatments wherever possible.
